# Relationship between perfluoroalkyl substance exposure and immunoglobulin E among Korean adults from Korean National Environmental Health Survey cycle 4 (2018–2020): a cross-sectional study

**DOI:** 10.3389/fpubh.2026.1761049

**Published:** 2026-03-31

**Authors:** Dongwoo Kim, Kyung-Jae Lee, June-Hee Lee, InHo Lee

**Affiliations:** 1 Department of Occupational and Environmental Medicine, Soonchunhyang University Seoul Hospital, Seoul, Republic of Korea; 2 Department of Occupational and Environmental Medicine, Soonchunhyang University Cheonan Hospital, Cheonan, Republic of Korea; 3 Department of Physiology, College of Medicine, Soonchunhyang University, Cheonan, Republic of Korea

**Keywords:** allergy, immunoglobulin E (IgE), Korean National Environmental Health Survey (KoNEHS), per- and polyfluoroalkyl substances (PFAS), sex difference

## Abstract

**Introduction:**

The potential immunotoxicity of per- and polyfluoroalkyl substances (PFAS) is a growing public health concern, yet epidemiological evidence in adults remains inconsistent. This inconsistency may partly stem from relying on single-pollutant models that do not fully capture the dynamics of chemical mixtures.

**Methods:**

We investigated the association between concurrent exposure to a mixture of five PFAS congeners and serum total immunoglobulin E (IgE) levels in a representative sample of 2,987 Korean adults from the 4th Korean National Environmental Health Survey (2018-2020). We employed Weighted Quantile Sum (WQS) regression, Quantile g-computation (Qgcomp), and Restricted Cubic Spline (RCS) analysis to assess mixture effects and non-linear associations.

**Results:**

A statistically significant positive association was identified between the overall PFAS mixture and total IgE levels (*β* = 0.033, *p* = 0.026), with perfluorodecanoic acid (PFDeA) being the major contributor. RCS analysis revealed a distinct J-shaped, non-linear dose-response relationship between PFDeA and IgE (P*_non-linear_* < 0.001). Furthermore, a significant interaction with smoking status was identified (*p* < 0.001); the positive association was significant only among non-smokers.

**Discussion/Conclusion:**

Exposure to long-chain PFAS mixtures is positively associated with atopic tendencies in adults. These findings highlight the importance of mixture modeling and lifestyle stratification in environmental epidemiology.

## Introduction

Allergic diseases continue to pose a substantial health burden globally, with high prevalence rates affecting millions of individuals across all age groups (1). Immunoglobulin E (IgE) plays a pivotal role in the pathogenesis of allergic diseases, and the serum total IgE concentration is widely used as a key indicator for predicting allergic sensitization and the risk of allergic disease (2). IgE binds to the FcεRI receptor on mast cells and basophils, triggering a degranulation response upon antigen exposure. This process promotes the release of histamine and cytokines, producing allergic symptoms (3). Identifying the environmental and genetic factors that cause changes in IgE levels is crucial for preventing and managing allergic diseases.

Among recent environmental exposure factors, perfluoroalkyl and polyfluoroalkyl substances (PFAS) have received particular attention. PFAS have been widely used in various industrial and consumer products such as food packaging, cookware, waterproof clothing, fire extinguishers, and electronics due to their water-, oil-, and heat-resistant properties (4, 5). Specifically, longer-chain PFAS (such as PFDeA) and other congeners like PFHxS are known to have exceptionally long half-lives in human blood. Generally, PFAS are known as “forever chemicals” because they barely undergo natural degradation and tend to persist in the environment and human body for extended periods. The general population is chronically exposed to PFAS through contaminated drinking water, seafood consumption, dust, and household products, leading to widespread detection of PFAS in people’s blood worldwide (6–9).

The impacts of PFAS on the human body include endocrine disruption, liver toxicity, and potential immunosuppression. Their relationship with the immune system has been a major concern, and a recent National Academies of Sciences, Engineering, and Medicine (NASEM) report classified PFAS as ‘immunotoxic’ chemicals (10). Several studies on children have reported that higher PFAS concentrations are associated with reduced vaccine antibody responses and a higher risk of asthma and atopy (11, 12). However, epidemiological evidence in adults remains inconsistent. While earlier studies reported positive associations (13, 14), recent investigations have highlighted the complexity of these relationships, suggesting potential non-linear dynamics or mixture-specific toxicity (15, 16). Conversely, others have shown conflicting results, including inverse or null associations (17). Most previous studies have relied on single-pollutant linear models, potentially overlooking the “cocktail effects” of co-occurring PFAS mixtures and non-linear dose–response relationships. These methodological limitations may account for the inconsistent findings reported in the literature.

Emerging epidemiological evidence suggests that PFAS may exhibit sex-specific immunological associations (18). Physiological factors unique to females, including menstruation, pregnancy, and hormonal fluctuations, significantly alter PFAS toxicokinetics, which may drive the observed sex-based heterogeneity in IgE production (19). These findings underscore the necessity of treating sex as a critical effect modifier in environmental exposure studies. Furthermore, lifestyle factors such as smoking and obesity modulate both immune function and PFAS toxicokinetics, acting as crucial confounders and effect modifiers (20, 21). Given that smoking inherently triggers systemic inflammation, it may profoundly alter baseline immunological profiles, highlighting the need to carefully stratify by such lifestyle factors to unmask true environmental associations. Elucidating the relationship between PFAS and IgE is therefore fundamental to understanding the pathogenesis of allergic diseases, and managing PFAS exposure represents a critical strategy for secondary prevention (22).

Therefore, this study evaluated the association between blood concentrations of major PFAS—including perfluorooctane sulfonate (PFOS), perfluorooctanoic acid (PFOA), perfluorononanoic acid (PFNA), perfluorodecanoic acid (PFDeA), and perfluorohexane sulfonate (PFHxS)—and total IgE concentrations in the general Korean adult population (*N* = 2,987) who participated in the 4th Korean National Environmental Health Survey (KoNEHS, 2018–2020). Unlike previous studies, we employed advanced statistical methods, including Weighted Quantile Sum (WQS) and Quantile g-computation (Qgcomp) regressions to comprehensively assess mixture associations, alongside Restricted Cubic Spline (RCS) analysis to explore non-linear dose–response relationships. Specifically, this study aimed to systematically investigate the association of PFAS mixture exposure with allergic sensitization and to elucidate potential susceptibilities related to sex and lifestyle factors, providing robust evidence for future environmental health policies.

## Materials and methods

### Study population

**Figure 1:**
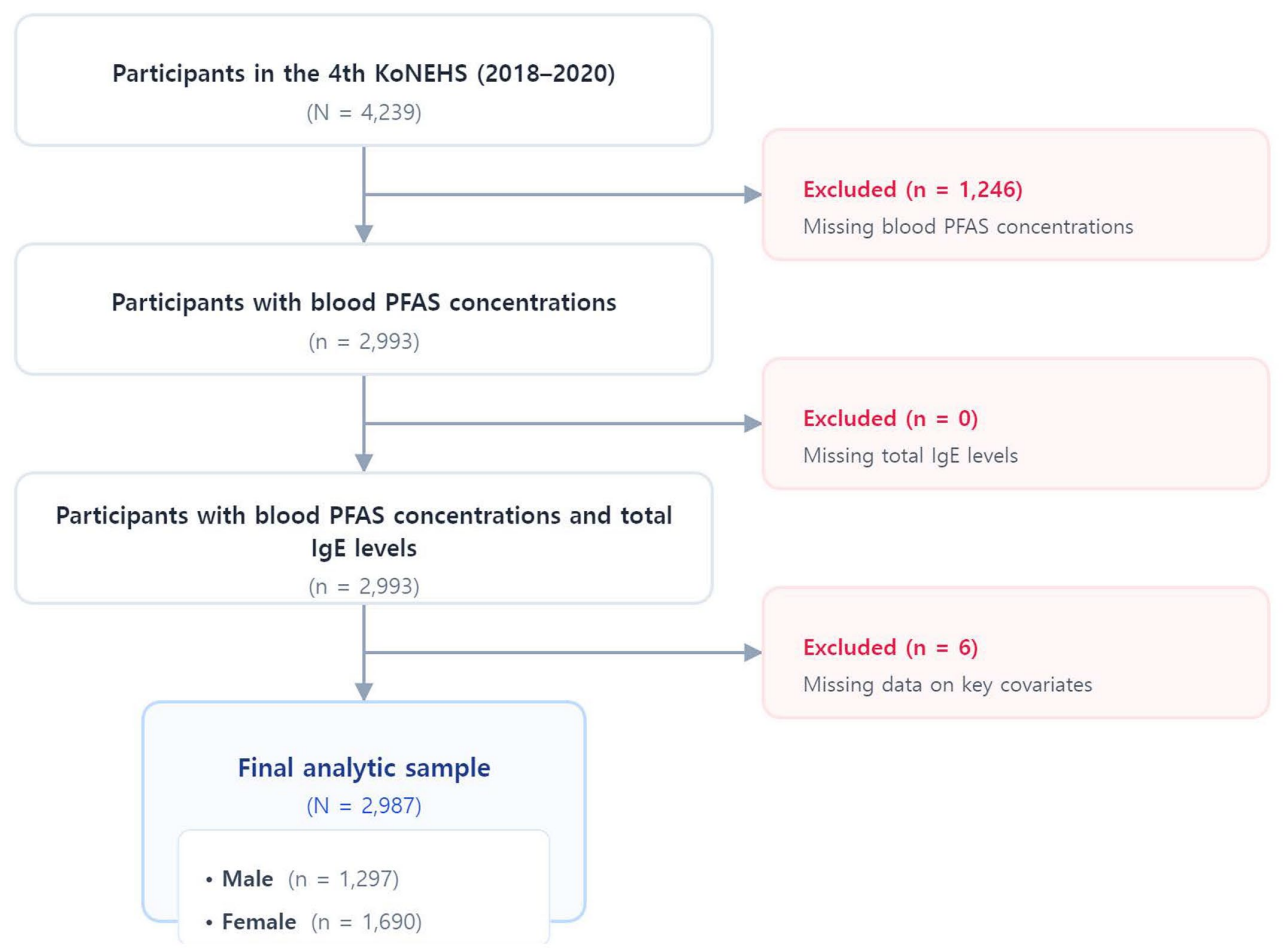
Flow diagram of study participant selection from the Fourth Korean National Environmental Health Survey (KoNEHS) 2018–2020.

This study utilized data from the fourth cycle of the Korean National Environmental Health Survey (KoNEHS), conducted between 2018 and 2020. The KoNEHS is a nationally representative biomonitoring survey designed to assess environmental exposure and health status in the general Korean population. Initially, 4,239 adults aged 19 years and older participated in the survey. We sequentially excluded individuals with missing data on blood PFAS concentrations (*n* = 1,246), missing total IgE levels (*n* = 0), and missing data on essential covariates (*n* = 6). Consequently, a final analytic sample of 2,987 participants was included in this cross-sectional study (Figure 1).

### Measurement of PFAS

This study utilized blood PFAS concentrations measured during the 4th KoNEHS. Whole blood samples were centrifuged to separate serum, which was then quantified using high-performance liquid chromatography–tandem mass spectrometry (HPLC-MS/MS). All analyses were conducted by a national environmental health analysis agency following standardized quality control (QC) and quality assurance (QA) procedures (23, 24). Values below the limit of detection (LOD) were replaced with half the LOD value.

### Data analysis

All statistical analyses were performed using R software, version 4.3.2 (R Foundation for Statistical Computing, Vienna, Austria). To ensure national representativeness, we utilized the blood sampling weights provided by the KoNEHS. Importantly, to prevent the inflation of statistical power and ensure accurate standard error estimation in regression models, sample weights were rescaled to sum to the final analytical sample size (*N* = 2,987). Due to right-skewed distributions, blood PFAS concentrations, total IgE levels, urinary cotinine, and creatinine were natural log-transformed (ln) prior to analysis to approximate normality. Extreme outliers were not explicitly excluded but were managed through the robust nature of the quantile-based approaches and log-transformation. Descriptive statistics for baseline characteristics were presented as medians with interquartile ranges (IQR) for non-normally distributed continuous variables, and as frequencies with percentages for categorical variables. Differences between groups (e.g., by sex) were evaluated using the Wilcoxon rank-sum test for continuous variables and the Chi-square test for categorical variables.

Prior to evaluating mixture associations, conventional single-pollutant multivariable linear regression models were fitted to estimate the independent association of each PFAS congener with total IgE. Subsequently, we calculated Variance Inflation Factors (VIF) to assess multicollinearity. Given the high multicollinearity observed (VIF > 20 for some congeners), we employed Weighted Quantile Sum (WQS) regression to evaluate the combined association of the PFAS mixture (25). We prioritized WQS over other mixture methods (e.g., BKMR) due to our *a priori* hypothesis regarding the unidirectional (adverse) association of PFAS and the method’s ability to provide interpretable empirical weights for individual congeners (26). The data were randomly split into training and validation sets. To ensure the stability of the weights, 1,000 bootstrap samples were used, and random seeds were established prior to analysis to ensure reproducibility. The models were comprehensively adjusted for age, sex, body mass index (BMI), log-transformed urinary cotinine, log-transformed serum and urinary creatinine, household income, education level, and alcohol consumption. Socioeconomic and behavioral variables, including household income (categorized as < 3, 3–5, and ≥ 5 million KRW/month), education level (≤ high school or > high school), and alcohol consumption (yes or no), were obtained from self-reported questionnaires. We utilized urinary cotinine as a continuous covariate to strictly adjust for recent nicotine exposure, while self-reported smoking status was used for subgroup stratification to capture long-term behavioral patterns. The WQS analysis was conducted using the gWQS package in R (27).

To further explore potential non-linear dose–response relationships for the predominant contributor identified in the WQS model (PFDeA), we utilized Restricted Cubic Spline (RCS) regression. We modeled the association using four knots located at the 5th, 35th, 65th, and 95th percentiles, and performed an Analysis of Variance (ANOVA) to test for non-linearity using the rms package (28). To formally validate the specific inflection point identified in the RCS curve, we subsequently stratified the study population into low- and high-exposure groups based on this threshold. Multivariable linear regression analyses, adjusted for all covariates, were then conducted to estimate and compare the associations within each respective stratum.

Subgroup analyses were performed by sex, smoking status (self-reported), and obesity status (BMI ≥ 25 kg/m^2^). To formally evaluate the modifying role of smoking, an interaction term (WQS index x smoking status) was introduced into the overall model. Furthermore, to untangle potential gender confounding driven by the high prevalence of smoking in men, we conducted a male-specific stratified analysis comparing current smokers and non-smokers.

Finally, rigorously designed sensitivity analyses were conducted. First, to address the limitation of the WQS regression—which assumes a unidirectional effect of all mixture components—we applied a Quantile g-computation (Qgcomp) model (29). Qgcomp relaxes this assumption, allowing for the simultaneous evaluation of bidirectional effects (both positive and negative weights) within the mixture. Second, to evaluate the clinical and epidemiological relevance of our findings, we applied logistic WQS regression using varying cut-off values for High IgE. We primarily used a baseline epidemiological cut-off of ln IgE ≥ 4.0 (approx. 54.6 IU/mL) to capture subclinical atopic tendencies, alongside higher clinical thresholds (≥ 100 IU/mL and ≥ 150 IU/mL) to verify the robustness of the results. Statistical significance was set at a two-sided *p*-value < 0.05.

### Ethics statement

This study was approved by the National Institute of Environmental Research (Management Number: NIER-2020-01-01-016). All data obtained in this study were anonymized, and the study adhered to the ethical principles of the 2013 Declaration of Helsinki by the World Medical Association.

## Results

### General characteristics of study participants

The general characteristics of the 2,987 participants included in the final analysis are summarized in Table 1. The study population consisted of 1,297 males (43.4%) and 1,690 females (56.6%), with a mean age of 52.5 years. The mean body mass index (BMI) was 25.0 kg/m^2^, and 15.6% of the participants were classified as current smokers based on self-reported questionnaires. Significant differences between sexes were observed in BMI, smoking prevalence, education level, alcohol consumption, and all measured biomarkers (all *p* < 0.001), except for age and household income. Specifically, males exhibited significantly higher levels of total IgE, serum creatinine, log-transformed urinary cotinine, and all five evaluated PFAS congeners compared to females (see Table 1).

**Table 1:** General characteristics of study participants (*N* = 2,987).

Variable	Total (*N* = 2,987)	Male (*N* = 1,297)	Female (*N* = 1,690)	*p*-value
Age (years), Mean (SD)	52.51 (14.70)	52.98 (15.09)	52.14 (14.39)	0.123
BMI (kg/m^2^), Mean (SD)	25.04 (3.65)	25.52 (3.46)	24.68 (3.75)	< 0.001
Current Smoker, *n* (%)	465 (15.6%)	419 (32.3%)	46 (2.7%)	< 0.001
Income, *n* (%)				0.328
Low	1,351 (45.2%)	571 (44.0%)	780 (46.2%)	
Middle	799 (26.7%)	364 (28.1%)	435 (25.7%)	
High	837 (28.0%)	362 (27.9%)	475 (28.1%)	
Education (≤ High school), *n* (%)	1,703 (57.0%)	680 (52.4%)	1,023 (60.5%)	< 0.001
Alcohol consumption, *n* (%)	2,350 (78.7%)	1,153 (88.9%)	1,197 (70.8%)	< 0.001
Biomarkers				
Total IgE (IU/mL), Median [IQR]	59.80 [21.90, 163.50]	97.90 [37.20, 268.00]	39.95 [15.72, 105.00]	< 0.001
Serum Creatinine (mg/dL), Mean (SD)	0.79 (0.20)	0.92 (0.17)	0.69 (0.17)	< 0.001
Urinary Cotinine (ng/mL), Median [IQR]	2.16 [0.81, 7.97]	3.39 [1.30, 608.47]	1.63 [0.57, 4.13]	< 0.001
PFAS Concentrations (μg/L)				
PFOA, Median [IQR]	7.15 [4.86, 10.17]	7.55 [5.34, 10.52]	6.72 [4.57, 9.82]	< 0.001
PFOS, Median [IQR]	17.10 [11.18, 25.54]	17.98 [12.25, 27.46]	16.37 [10.42, 23.86]	< 0.001
PFNA, Median [IQR]	2.45 [1.58, 3.66]	2.65 [1.78, 3.87]	2.30 [1.43, 3.49]	< 0.001
PFDeA, Median [IQR]	1.04 [0.70, 1.50]	1.12 [0.74, 1.60]	0.99 [0.67, 1.41]	< 0.001
PFHxS, Median [IQR]	4.33 [2.74, 6.95]	4.97 [3.29, 7.73]	3.87 [2.40, 6.33]	< 0.001

SD, standard deviation; GM, geometric mean; CI, confidence interval; PFAS, per- and polyfluoroalkyl substances; IgE, immunoglobulin E.

^*^Normally distributed continuous variables are presented as mean (standard deviation), while non-normally distributed continuous variables (Total IgE, urinary cotinine, and PFAS) are presented as median [interquartile range]. Differences between sexes were tested using independent *t*-tests or Wilcoxon rank-sum tests for continuous variables, and chi-square tests for categorical variables.

### Single-pollutant associations and WQS regression analysis

Prior to the mixture analysis, we evaluated the individual associations between each PFAS and total IgE levels using conventional single-pollutant multivariable linear regression models, adjusted for all covariates (Supplementary Table S1). In these single-pollutant models, only PFDeA demonstrated a statistically significant positive association with total IgE levels (*β* = 0.178, 95% CI: 0.068, 0.288; *p* = 0.001). PFOS and PFNA showed non-significant positive associations, while PFOA and PFHxS exhibited non-significant negative directions.

**Figure 2:**
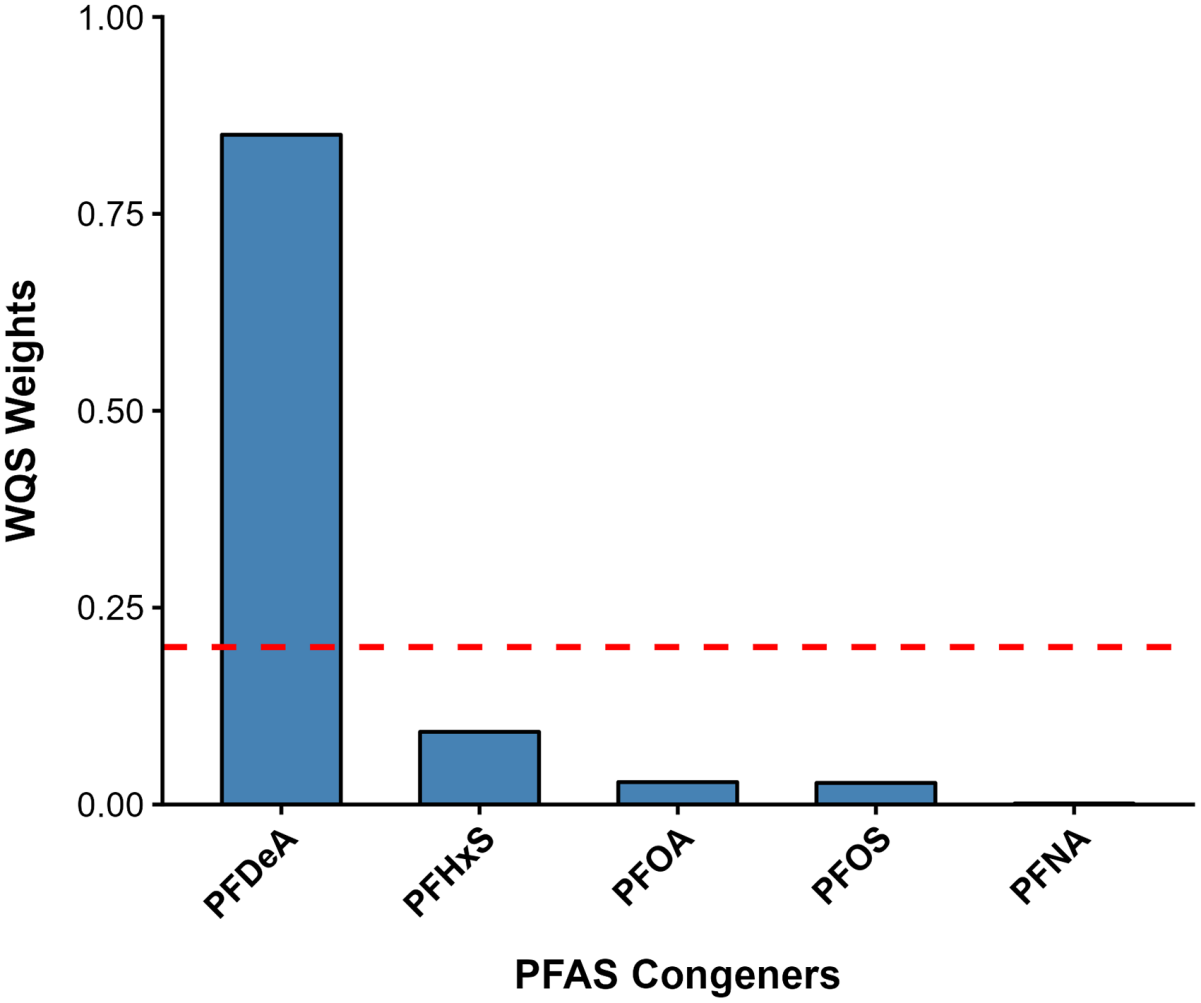
Contribution weights of individual PFAS congeners to the WQS index targeting total IgE.

To evaluate the combined effect of the PFAS mixture while addressing multicollinearity, a WQS regression analysis was performed (Table 2). The WQS index representing the overall PFAS mixture was significantly and positively associated with total IgE levels (β = 0.033, 95% CI: 0.004, 0.062; *p* = 0.026), after adjusting for age, sex, BMI, log-transformed urinary cotinine, log-transformed serum and urinary creatinine, household income, education level, and alcohol consumption. Among the five PFAS congeners, PFDeA was the overwhelmingly dominant contributor to the mixture effect (Figure 2), which aligns consistently with the single-pollutant model findings.

**Table 2:** Association between the weighted quantile sum (WQS) index of PFAS mixture and total IgE concentrations (*N* = 2,987).

Variable	Coefficient (β)	Std. Error (SE)	*t*-value	*p*-value
WQS index (mixture effect)	0.033	0.015	2.23	0.026*
Covariates				
Age	−0.004	0.003	−1.24	0.213
Sex (female)	−0.677	0.095	−7.13	< 0.001*
BMI	0.021	0.009	2.36	0.019*
Urinary cotinine (ln)	0.051	0.013	4	< 0.001*
Serum creatinine (ln)	0.257	0.202	1.27	0.204
Urinary creatinine (ln)	0.013	0.049	0.27	0.785
Income (middle)	−0.162	0.084	−1.94	0.052
Income (high)	−0.127	0.085	−1.5	0.135
Education (≤ high school)	−0.129	0.08	−1.6	0.109
Alcohol consumption (yes)	0.164	0.085	1.93	0.054

The WQS regression model was adjusted for age, sex, body mass index (BMI), urinary cotinine (log-transformed), serum and urinary creatinine (log-transformed), household income, education level, and alcohol consumption. *p < 0.05.

### Non-linear dose–response relationship

**Figure 3:**
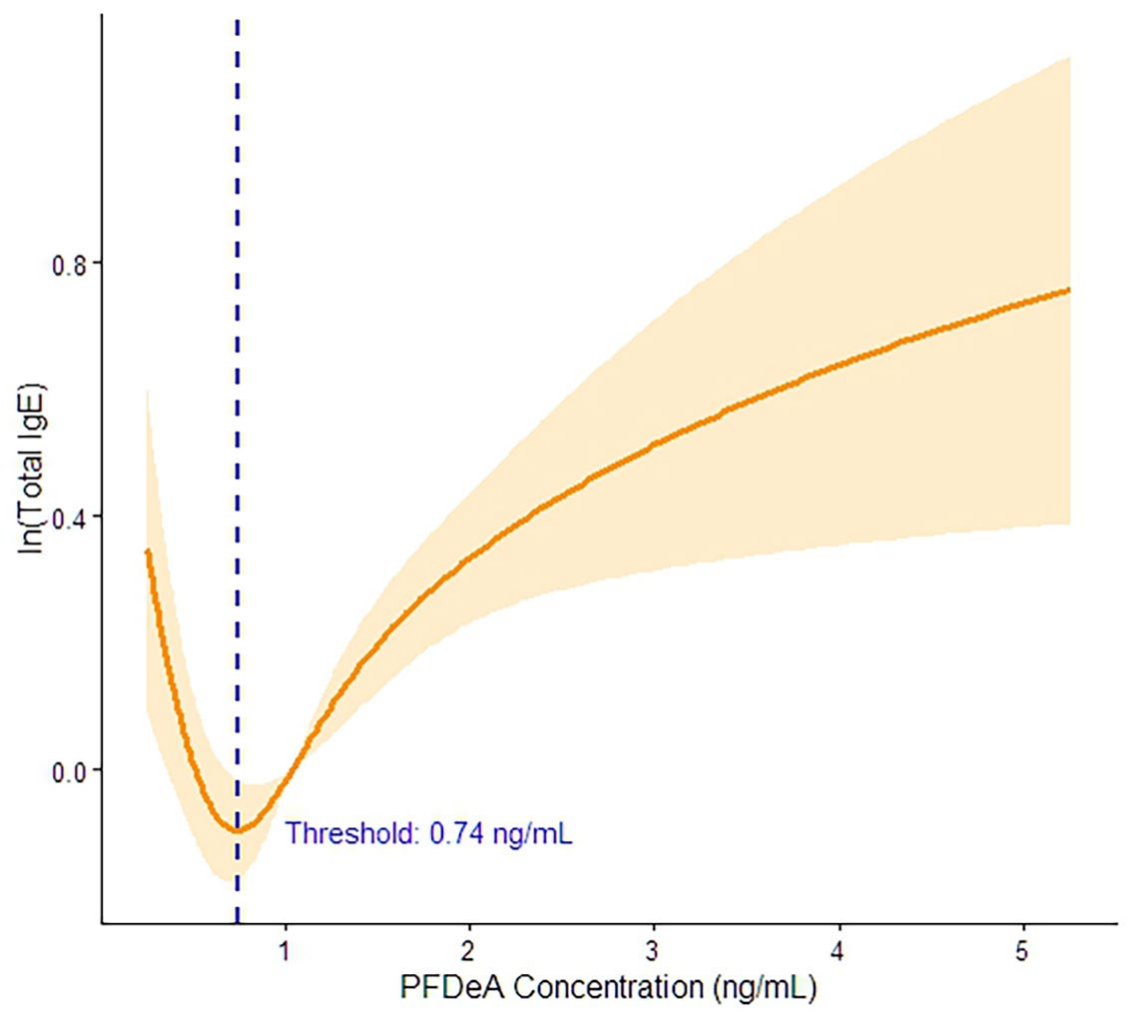
Dose–response relationship between ln-transformed PFDeA and ln-transformed total IgE using restricted cubic splines (RCS).

To further investigate the potential non-linear dose–response relationship between the predominant contributor (PFDeA) and total IgE, we conducted a restricted cubic spline (RCS) regression analysis (Figure 3 and Supplementary Table S2). The RCS model, adjusted for all covariates, revealed a highly significant non-linear, J-shaped relationship (P*_non-linear_* < 0.001). Specifically, total IgE levels remained relatively flat at lower concentrations but began to exhibit an upward trend once the blood PFDeA concentration exceeded the critical threshold of 0.74 ng/mL (corresponding to a natural log-transformed value of −0.30). To formally validate this non-linear dose–response, we stratified the participants into low-exposure (< 0.74 ng/mL, *N* = 854) and high-exposure (≥ 0.74 ng/mL, *N* = 2,133) groups. In multivariable linear regression models adjusted for all covariates, no significant association was observed in the low-exposure group (*β* = −0.350, 95% CI: −0.720, 0.020; *p* = 0.065). In contrast, a highly significant positive association was confirmed in the high-exposure group (*β* = 0.399, 95% CI: 0.244, 0.554; *p* < 0.001), statistically supporting the threshold-dependent relationship.

### Subgroup analyses

Stratified WQS analyses were performed to identify potentially susceptible subgroups (Table 3). While the positive association between the PFAS mixture and total IgE was significant in males (*β* = 0.076, *p* = 0.002) and non-smokers (*β* = 0.037, *p* = 0.037), no significant associations were observed in females or current smokers.

**Table 3:** Stratified analyses of the association between PFAS mixture and total IgE by sex, smoking status, and obesity.

Subgroup	*N*	Coefficient (95% CI)	*p*-value
Sex			
Male	1,297	0.076 (0.027, 0.125)	0.002*
Female	1,690	−0.016 (−0.053, 0.021)	0.392
Smoking status			
Current smoker	465	0.071 (−0.013, 0.155)	0.098
Non-smoker	2,522	0.037 (0.002, 0.072)	0.037*
Interaction (WQS × smoking)		–	< 0.001*
Obesity status			
Obese (BMI ≥ 25)	1,412	0.020 (−0.025, 0.065)	0.387
Normal (BMI < 25)	1,575	0.037 (−0.008, 0.082)	0.105
Male-specific analysis			
Male smoker	419	0.063 (−0.015, 0.141)	0.118
Male non-smoker	878	0.078 (0.023, 0.133)	0.005*

Models were adjusted for relevant covariates (age, sex, BMI, urinary cotinine, serum and urinary creatinine, household income, education level, and alcohol consumption) corresponding to each subgroup. Current smokers were defined based on self-reported smoking status. Obesity was defined as a BMI ≥ 25 kg/m^2^. CI, Confidence interval. *p < 0.05.

To formally evaluate the modifying effect of smoking, we tested for an interaction between the WQS index and smoking status in the overall sample, yielding a highly significant interaction (*p* < 0.001). Furthermore, recognizing the substantially higher smoking prevalence in men, we conducted a male-specific stratified analysis to determine whether this smoking effect was independent of gender confounding. Interestingly, within the male subgroup, the positive association was robust and statistically significant only in male non-smokers (*β* = 0.078, 95% CI: 0.023, 0.133; *p* = 0.005), whereas it was attenuated and non-significant in male smokers (β = 0.063, 95% CI: −0.015, 0.141; *p* = 0.118) (see Table 3).

**Table 4:** Sensitivity analysis using logistic regression models for high total IgE levels with varying cut-offs.

Outcome Definition	Odds ratio (95% CI)	*p*-value
High IgE (ln IgE ≥ 4.0)	1.020 (0.972, 1.072)	0.418
High IgE (IgE ≥ 100 IU/mL)	1.014 (0.970, 1.060)	0.54
High IgE (IgE ≥ 150 IU/mL)	1.028 (0.979, 1.079)	0.27

The binary outcomes were defined as follows: High IgE (ln IgE ≥ 4.0, approx. 54.6 IU/mL), High IgE (≥ 100 IU/mL), and High IgE (≥ 150 IU/mL). All models were adjusted for age, sex, BMI, urinary cotinine, serum and urinary creatinine, household income, education level, and alcohol consumption. CI, Confidence interval.

## Discussion

This study evaluated the association between exposure to a mixture of five perfluoroalkyl substance (PFAS) congeners and total IgE levels in Korean adults (*N* = 2,987). By employing Weighted Quantile Sum (WQS) regression and Restricted Cubic Spline (RCS) analysis, we addressed the limitations of traditional single-pollutant models and provided comprehensive insights into mixture associations and non-linear dose–response relationships. Our analytical approach aligns with current trends in environmental epidemiology, where sophisticated mixture modeling is increasingly adopted to identify key drivers within chemical mixtures while addressing multicollinearity (16, 30).

The WQS index showed a statistically significant positive association with total IgE levels (*β* = 0.033, *p* = 0.026) after adjusting for comprehensive covariates, including newly incorporated socioeconomic and behavioral factors. A notable finding was the major contribution of PFDeA, which accounted for the vast majority of the positive mixture weight. This result suggests a potential shift in the primary immunomodulatory correlates from legacy compounds (PFOS/PFOA) to longer-chain PFAS like PFDeA. Long-chain PFAS are known to have longer biological half-lives and higher affinity for plasma proteins than shorter-chain analogs (31), potentially leading to greater bioaccumulation (4, 32). Mechanistically, recent evidence suggests that PFAS exposure is linked to altered profiles of pro-inflammatory cytokines. Specifically, higher PFAS exposure has been observationally correlated with elevated levels of IL-4, a key Th2 cytokine that stimulates B-cells to synthesize IgE (33). This is consistent with experimental models proposing that PFAS may be associated with Th2-skewed immune responses via PPAR-α signaling modulation and altered fatty acid metabolism (34). Furthermore, our results are in line with recent epidemiological data indicating that serum PFDeA is associated with asthma exacerbations in the US population (15).

Consistent with our findings, previous reviews (12) and epidemiological studies (35, 36) have reported positive associations between PFAS exposure and allergic outcomes. However, our research provides a more nuanced understanding by identifying PFDeA as the predominant driver and revealing a distinct threshold-dependent relationship. The RCS analysis revealed a significant nonlinear J-shaped relationship between PFDeA and total IgE (Figure 3, P*_non-linear_* < 0.001). The curve remained stable at lower concentrations but exhibited an upward trend once blood PFDeA levels exceeded the critical threshold of 0.74 ng/mL. This non-monotonic dose–response (NMDR) aligns with the established properties of endocrine-disrupting chemicals, where complex low-dose dynamics cannot be simply extrapolated from high-dose models (37). This J-shaped pattern suggests that the potential promotion of Th2-skewing associated with PFDeA may be an accumulation-dependent process, requiring specific thresholds to surpass biological compensatory mechanisms (38). Furthermore, while most domestic studies in Korea have focused on non-immunological outcomes (39–41) or pediatric populations (42), our study fills a critical gap by demonstrating a significant association in the general adult population.

An important finding of our study emerged from the subgroup and interaction analyses regarding smoking status and sex. We observed a statistically significant interaction between the WQS index and smoking status (*p* < 0.001). Interestingly, the stratified analysis revealed that the positive association between the PFAS mixture and total IgE was significant only in non-smokers, particularly male non-smokers (*β* = 0.078, *p* = 0.005), whereas it was attenuated and non-significant in current smokers. This divergence may be explained by a potential ‘masking effect.’ Smoking is inherently a powerful trigger for systemic inflammation and IgE elevation. Consequently, the baseline IgE levels in smokers are already substantially elevated, which may overshadow or mask the independent, comparatively subtle contribution of PFAS exposure. In contrast, in the absence of the pro-inflammatory stimulus of smoking, the distinct positive association between PFAS (primarily PFDeA) and IgE elevation becomes more apparent. While previous studies have hypothesized a ‘double-hit’ mechanism where smoking and PFAS concurrently compromise epithelial barriers (21, 43, 44), our population-level data suggests that baseline inflammatory ceilings may play a more complex masking role, highlighting the need for careful stratification in future environmental epidemiology studies.

Regarding BMI, we did not observe a statistically significant modifying effect of obesity. Furthermore, to ensure the robustness of our findings, we conducted rigorous sensitivity analyses. First, applying a Quantile g-computation (Qgcomp) model revealed a positive, albeit statistically non-significant, overall mixture effect on total IgE (*ψ* = 0.021, 95% CI: −0.006, 0.048; *p* = 0.126). This attenuation in overall significance reflects the bidirectional nature of the Qgcomp model, where the net effect was diluted by the counteracting negative weights of other congeners (e.g., PFHxS). Crucially, however, evaluating the specific directional contributions demonstrated that PFDeA accounted for an overwhelming 98.5% of the positive weights. This confirms that the positive immunomodulatory direction within the mixture is almost exclusively driven by PFDeA, thereby reinforcing the validity of our WQS findings. Second, when converting the continuous total IgE into dichotomous outcomes (e.g., ln IgE ≥ 4.0), the associations trended positively but did not reach statistical significance (see Table 4). This implies that while higher PFAS exposure is associated with an upward shift in the overall distribution of IgE levels, it may not be the sole determinant pushing individuals across strict clinical diagnostic thresholds in a general, non-clinical population.

This study has several strengths. First, sophisticated mixture models (WQS, Qgcomp, and RCS) were applied to investigate PFAS-IgE associations in a representative Korean adult population. Second, we minimized potential statistical bias by rigorously adjusting for comprehensive confounders, including socioeconomic factors, cotinine, and creatinine. Third, we explored crucial modifying factors, unveiling the masking effect of smoking.

However, some limitations must be acknowledged. First, due to the cross-sectional design, we cannot establish temporal relationships or rule out reverse causation, where physiological states related to allergic inflammation might influence PFAS metabolism and excretion rates. Nevertheless, there is currently no established physiological mechanism to suggest that allergic inflammation significantly alters the toxicokinetics or renal excretion of PFAS. Therefore, the forward direction—where PFAS exposure exacerbates a Th2-skewed immune response—remains biologically more plausible. Second, although we adjusted for major confounders, potential residual confounding from unmeasured factors—such as personal history of atopic diseases, occupational exposure, or co-exposure to indoor allergens—cannot be excluded. Furthermore, due to the inherent constraints of the secondary KoNEHS data, we could not adjust for detailed female-specific physiological factors that influence PFAS toxicokinetics, such as menstruation, parity, or oral contraceptive use. Third, we analyzed total IgE, a nonspecific indicator of atopic tendency, rather than allergen-specific IgE or clinical allergic disease diagnoses. Finally, while we incorporated both urinary cotinine and self-reported questionnaires to validate smoking status, single-spot biological samples may not perfectly reflect long-term exposure trajectories.

## Conclusion

In conclusion, our study demonstrates a notable positive association between exposure to a PFAS mixture and total IgE levels in the general Korean adult population. This association was primarily driven by the long-chain congener PFDeA, which exhibited a distinct non-linear, J-shaped dose–response relationship characterized by a specific threshold. Furthermore, we identified a potential masking effect of smoking, revealing that the positive association between PFAS exposure and IgE elevation is more apparent in the absence of smoking-induced baseline inflammation. These findings highlight the importance of employing sophisticated mixture modeling and carefully considering lifestyle modifiers in environmental epidemiology. Further prospective studies are warranted to confirm these observational findings and elucidate the underlying immunological mechanisms of long-chain PFAS. Building upon these findings in the general population, future studies should extend this framework to highly exposed occupational cohorts—such as firefighters handling aqueous film-forming foams (AFFF) or chemical workers—to further elucidate the immunotoxic impacts of PFAS.

## Data Availability

Publicly available datasets were analyzed in this study. This data can be found at The Korean National Environmental Health Survey (KoNEHS), https://www.ehtis.or.kr/.
