# Supplementary material for: Relationship between perfluoroalkyl substance exposure and immunoglobulin E among Korean adults from Korean National Environmental Health Survey cycle 4 (2018–2020): a cross-sectional study
Source: Front Public Health. 2026 Mar 31;14:1761049. doi: 10.3389/fpubh.2026.1761049 (PMC13076250; doi:10.3389/fpubh.2026.1761049)
Supplement: Supplementary file 1 [file Data_Sheet_1.docx]

**Supplementary Materials**

**Supplementary Table S1. Single-pollutant multivariable linear regression models for the association between individual PFAS congeners and total IgE**

| **PFAS Congener** | **Coefficient (β) (95% CI)** | **P-value** |
| --- | --- | --- |
| **ln(PFOS)** | **0.044 (-0.054, 0.142)** | **0.383** |
| **ln(PFOA)** | **-0.005 (-0.107, 0.097)** | **0.916** |
| **ln(PFNA)** | **0.090 (-0.012, 0.192)** | **0.083** |
| **ln(PFDeA)** | **0.178 (0.068, 0.288)** | **0.001*** |
| **ln(PFHxS)** | **-0.063 (-0.136, 0.010)** | **0.087** |

Each PFAS congener was evaluated in a separate multivariable linear regression model. All models were adjusted for age, sex, BMI, ln-cotinine, ln-serum creatinine, ln-urinary creatinine, household income, education level, and alcohol consumption. CI: Confidence interval. * P < 0.05.

**Supplementary Table S2. Analysis of Variance (ANOVA) for the Restricted Cubic Spline (RCS) regression model evaluating the non-linear association of PFDeA**

| **Source** | **d.f.** | **Partial SS** | **Mean Square (MS)** | **F-statistic** | **P-value** |
| --- | --- | --- | --- | --- | --- |
| **ln(PFDeA) (Overall)** | **3** | **82.66** | **27.55** | **14.43** | **< 0.001*** |
| **Nonlinear component** | **2** | **62.86** | **31.43** | **16.46** | **< 0.001*** |
| **Age** | **1** | **5.12** | **5.12** | **2.68** | **0.102** |
| **Sex** | **1** | **161.29** | **161.29** | **84.47** | **< 0.001*** |
| **BMI** | **1** | **10.03** | **10.03** | **5.25** | **0.022*** |

The ANOVA table demonstrates a highly significant overall association for PFDeA, driven significantly by its non-linear component (P_non-linear_ < 0.001). Abbreviations: d.f., degrees of freedom; SS, sum of squares. * P < 0.05. Data for remaining covariates (cotinine, creatinine, socioeconomic and behavioral factors) are omitted for brevity but were included in the full model.

**Supplementary Figure S1. Positive and negative weights of individual PFAS congeners derived from the Quantile g-computation (Qgcomp) model.**

**
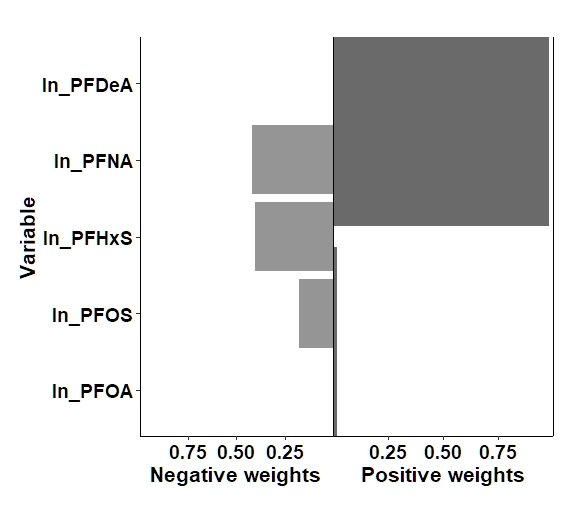
**

**Alt text / Legend:** A bar chart displaying the independent positive (right) and negative (left) weights of five PFAS congeners on total IgE levels, estimated using the Qgcomp model. Unlike the WQS regression, the Qgcomp model allows for bidirectional evaluations. While the overall mixture effect was attenuated (Ψ= 0.021, P = 0.126) due to the counteracting negative weights of PFNA, PFHxS, and PFOS, the positive weights were overwhelmingly dominated by **PFDeA (98.5%)**. This finding confirms that the predominant contribution of PFDeA to IgE elevation is highly robust, regardless of the methodological constraints regarding directionality. The model was adjusted for age, sex, BMI, ln-cotinine, ln-serum creatinine, ln-urinary creatinine, household income, education level, and alcohol consumption.
